# A Novel Mutation in the VPS13B Gene in a Cohen Syndrome Patient with Positive Antiphospholipid Antibodies

**DOI:** 10.1155/2021/3143609

**Published:** 2021-08-25

**Authors:** Roghayeh Dehghan, Mahdiyeh Behnam, Alireza Moafi, Mansoor Salehi

**Affiliations:** ^1^Department of Genetics and Molecular Biology, School of Medicine, Isfahan University of Medical Science, Isfahan, Iran; ^2^Cellular, Molecular and Genetics Research Center, Isfahan University of Medical Sciences, Isfahan, Iran; ^3^Pediatric Hematology and Oncology, Isfahan University of Medical Sciences, Isfahan, Iran; ^4^Pediatric Inherited Diseases Research Center, Research Institute for Primordial Prevention of Non-communicable Disease, Isfahan University of Medical Sciences, Isfahan, Iran; ^5^Child Growth and Development Research Center, Research Institute for Primordial Prevention of Non-Communicable Disease, Isfahan University of Medical Sciences, Isfahan, Iran

## Abstract

Cohen syndrome is an autosomal recessive disorder with the primary symptoms of mental deficiency, progressive retinopathy, hypotonia, microcephaly, obesity of midchildhood onset, intermittent neutropenia, and dysmorphic facial features. The syndrome has high phenotypic heterogeneity and is caused by loss-of-function mutations in the VPS13B gene. Here, we introduce a novel homozygous nonsense mutation (c.8698G > T, p.E2900X) in the VPS13B gene in an 11-year-old Iranian boy with major symptoms of Cohen syndrome. He also had mild anemia accompanied by positive antiphospholipid antibodies, the latter has never been previously reported in Cohen syndrome.

## 1. Introduction

Cohen syndrome is an autosomal recessive disorder that was first described by Cohen in 1973. The authors reported three Finnish cases with hypotonia, obesity of midchildhood onset, mental deficiency, and ocular-cranial anomalies [1]. Neutropenia was later identified as another continuous symptom of the disease, and the discovery of over 100 cases worldwide revealed Cohen syndrome to have high phenotypic heterogeneity, particularly in non-Finnish patients [2, 3].

In 2003, Cohen syndrome was shown to be caused by loss-of-function mutations in the gene VPS13B (also known as *COH1*; 4). This large gene, containing 62 exons, encodes a peripheral membrane protein composed of several domains and functional motifs. Cellular and molecular studies have shown that VPS13B is involved in vesicle‐mediated sorting and the intracellular transport of proteins [4–7]. However, it is not clear how and through which cellular pathways VPS13B mutations lead to multisystemic symptoms of Cohen syndrome. The identification and study of novel mutations could broaden our knowledge on the association between different symptoms of Cohen syndrome and different functional motifs of the VPS13B gene. This knowledge could, in turn, help further elucidate the cellular functions and pathways of VPS13B in organs involved in the pathology of Cohen syndrome.

In the present case-report study, we introduce a case of Cohen syndrome with a novel homozygous nonsense mutation (c.8698 G > T, p.E2900X) in exon 48 of the VPS13B gene. This case, in addition to the common major symptoms of the syndrome, also presented with positive antiphospholipid antibodies (APAs), which have not been previously reported in Cohen syndrome to date.

## 2. Case Presentation

### 2.1. Clinical Studies

An 11-year-old Iranian boy with syndromic obesity was referred to the Medical Genetics Centre of Genome (Isfahan, Iran) for genetic analysis. The child had mild mental retardation, hyperactivity, and progressive visual impairment, with signs of retinal pigmentary changes and waxy pale optic discs in eye examination. The patient had truncal obesity, long slender extremities, and joint hyperlaxity at the time of referral. According to his medical documents, although the child had a normal head circumference at birth, he began to develop microcephaly at the age of one. In addition, the patient had a history of developmental milestones, such as making his first steps later than is normal at age 19 months. His facial features included hypertelorism, thick eyebrows, thick bushy hair, a low hairline, and a short upper lip. The patient had been having frequent colds since infancy, and his complete blood count showed persistent moderate neutropenia and mild anemia, with an absolute neutrophil count (ANC) of 600 cells/uL and a hemoglobin level of 11.8 g/dL in his last hematological test at the time of referral. At age six, the child experienced thrombocytopenia-associated bleeding with a very low platelet count of 21,000/uL. However, the platelet count was in the normal range in further follow-up CBC tests. Following the aforementioned thrombocytopenic bleeding, antiphospholipid antibodies were evaluated, and the patient was found to be positive for two main APAs: anticardiolipin IgG and anti-*β*2-glycoprotein 1 IgG, with a titration of 23.7 and 300.2 U/mL, respectively, in his last APA test at age 11. However, the patient was negative for lupus anticoagulant and antinuclear antibodies (ANAs), and clinical examination ruled out systemic lupus erythematosus (SLE) and rheumatoid arthritis, which are two suspected underlying autoimmune diseases for positive antiphospholipid antibodies.

The proband was the only child of healthy consanguineous parents (first cousin), and similar symptoms had not been reported in any of his first- or second-degree relatives. These criteria suggested that he has an autosomal recessive genetic disorder. Cohen syndrome, in particular, was suspected based on his symptoms, and after obtaining informed consent of the parents, whole-exome sequencing was performed for genetic diagnosis.

### 2.2. Genetic Studies

**Figure 1:**
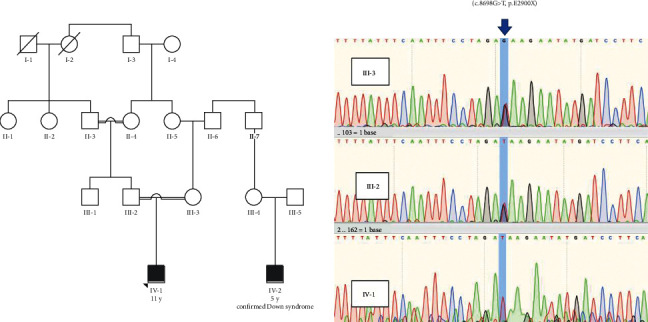
Sanger sequencing confirmed the homozygosity of the patient and heterozygosity of his parents for c.8698 G > T, p.E2900X in the VPS13B gene.

Genomic DNA was extracted from the proband's peripheral blood and subjected to clinical whole-exome sequencing at a depth of 100X on an Illumina HiSeq 4000. Sequence data were aligned to the human reference genome (GRCh38) using the Burrows–Wheeler Alignment Tool. Variants were called using the Genome Analysis Tool Kit and annotated using ANNOVAR. We detected a homozygous nonsense variant (c.8698 G > T, p.E2900X) in exon 48 of the VPS13 B gene and confirmed the homozygosity of the patient and heterozygosity of his parents for the variant using Sanger sequencing (Figure 1).

## 3. Discussion

Cohen syndrome is a genetic disorder with high phenotypic variability and primary symptoms of mental deficiency, progressive retinopathy, hypotonia, microcephaly, obesity of midchildhood onset, intermittent neutropenia, and dysmorphic facial features [3]. This case report describes the case of an 11-year-old boy who presented with typical symptoms of Cohen syndrome, as well as persistent mild anemia. In addition, serological tests revealed that the patient had been positive for anticardiolipin and anti-*β*2-glycoprotein 1 IgGs since the age of six when he was first tested for antiphospholipid syndrome (APS) following a thrombocytopenic bleeding complication. APS is an autoimmune disease characterized by the detection of circulating APAs and arterial/venous thrombosis. It is associated with recurrent miscarriage in pregnant women and thrombocytopenia in some patients [8]. APS can occur primarily, without association with other diseases or secondarily to other autoimmune disorders, such as SLE and rheumatoid arthritis. Medical examinations and records had ruled out the APS-related autoimmune disorders in our patient, and he had never experienced arterial/venous thrombosis.

To the best of our knowledge, to date, no study has evaluated the APS and APAs in Cohen syndrome. However, there is one report of thrombocytopenia in a male patient with a homozygous deletion of the first eight exons of the VPS13B gene [9, 10]. Interestingly, thrombocytopenia with a platelet count below <100 × 10^9^ L^−1^ and <150 × 10^9^ L^−1^ has been reported in 20% and 53% of APS patients, respectively, which has led to bleeding complications in rare cases [11, 12]. In addition, thrombosis, a main clinical manifestation of APS, has been reported in two siblings affected with symptoms of Cohen syndrome. Laboratory tests revealed a combined deficiency of protein C, protein S, and antithrombin III in these siblings, while their APAs were not evaluated [13]. Some studies have shown an interaction between APAs with coagulation proteins, such as protein C; for this reason, we suspect an underlying role of these antibodies in the coagulation defects reported in these siblings [14].

The positive APAs in our patient, as well as two previous reports of thrombocytopenia and thrombosis in Cohen syndrome, raise several questions. Could mutations in the VPS13B gene drive APA in some cases of Cohen syndrome? If so, could APAs contribute to rare instances of thrombocytopenia or thrombosis in Cohen syndrome? To answer these questions, it is necessary to study the medical history of thrombocytopenia and thrombosis in more cases of Cohen syndrome and evaluate the platelet count and APAs in these cases. These evaluations, beyond their research value, could also aid in risk assessments and in preventing hematological complications in Cohen patients.

In this case report, in addition to reporting a new pathogenic mutation in the VPS13B gene, we described positive APAs in a patient with Cohen syndrome for the first time. Future studies could investigate the relationship between Cohen syndrome and APAs and explore whether these antibodies could be the underlying cause of rare instances of thrombocytopenia or coagulation disorders in Cohen syndrome. Given that APAs can cause serious issues such as thrombosis and bleeding complications, they should be evaluated as a precautionary measure in Cohen patients.

## Data Availability

Sequencing results are available for the case on request.
